# Rapid monitoring of the target protein expression with a fluorescent signal based on a dicistronic construct in *Escherichia coli*

**DOI:** 10.1186/s13568-018-0612-5

**Published:** 2018-05-21

**Authors:** Chang-Ye Hui, Yan Guo, Wen Zhang, Xian-Qing Huang

**Affiliations:** Department of Pathology & Toxicology, Shenzhen Prevention and Treatment Center for Occupational Disease, Shenzhen, 518020 People’s Republic of China

**Keywords:** Recombinant protein expression, Dicistron, Fluorescent signal, Real-time, Quantification

## Abstract

Real-time quantification of recombinant proteins is important in studies on fermentation engineering, cell engineering, etc. Measurement of the expression level of heterologous proteins in bacterial fermentation broth has traditionally relied on time-consuming and labor-intensive procedures, such as polyacrylamide gel electrophoresis, immunoblot analysis, and biological activity assays. We describe a simple, fast, and high sensitive assay for detecting heterologous proteins production in bacteria either at the overall level (fluorescence spectrophotometry) or at the individual level (fluorescence microscopic image) in this study. Based on a dicistronic model, the translation of target gene in the upstream open reading frame (ORF) was coupled with the synthesis of the mCherry reporter in the downstream ORF in *E. coli* cells, and subsequently this demonstrated a positive correlation between the expression of target gene and mCherry. Although a time lag exists between the expression of target protein and mCherry reporter, the method described here allows facile monitoring of dynamic changes in target protein expression, relying on indirect determination of the fluorescence intensity of mCherry during fermentation in real-time models. Additionally, the performance of a single bacterial cell factory could be checked under the fluorescence microscope field.

## Introduction

It is a long time since *Escherichia coli* (*E. coli*) was developed as an ideal microbial cell factory for the production of recombinant proteins. The application of highly active bacteriophage RNA polymerase, especially T7 RNA polymerase, made the expression system very powerful for overproduction of recombinant proteins (Peranen et al. 1996). An *E. coli*-based expression system is certainly used as the preferred system, and numerous heterologous proteins of different origins have been successfully expressed in recombinant *E. coli* (Hui et al. 2018b; Mahalik et al. 2014).

The expression level of recombinant protein can be affected by many factors, including genetic elements, secondary structure of mRNA, codon preference, culture conditions, and more (Mahalik et al. 2014). Measurement of the expression level of recombinant proteins in fermentation broth has generally relied on time-consuming and labor-intensive routine assays such as PAGE, western blot, ELISA, and biological activity assay. Currently, there are few approaches to precisely detect the expression level of the target protein, not to mention the technique for real-time monitoring of the translation level of the recombinant protein.

Operons are multigene transcriptional units which occur primarily in prokaryotes but also in some eukaryotes (Blumenthal 2004; Jacob et al. 2005). A typical bacterial operon was defined as several structural genes arranged in tandem that are transcribed in a single polycistronic mRNA under the control of the same promoter (Jacob et al. 2005). However, naturally occurring genetic regulatory elements hardly meet the requirements for biotechnological applications. Progress in the development of artificial hybrid operons promises to greatly broaden the use of *E. coli* as a model for the production of heterologous proteins (Yadav et al. 2014).

In this paper, we have developed a molecular device in *E. coli* using a dicistronic construct. A short ORF containing multiple cloning sites (MCS) was placed upstream of another ORF encoding red fluorescence protein mCherry as reporter. The target gene can be ligated into MCS, and both the target gene and the reporter gene are co-transcribed into one dicistronic mRNA. More importantly, the expression of the target gene would also be coupled with the expression of the reporter mCherry. Thus, the target protein production could be easily quantitated by indirect determination of the fluorescence intensity of mCherry in crude *E. coli* culture in real-time. The fluorescent signal can be noninvasively detected in living *E. coli* cells during culture at any time. This eliminates the analysis of exogenously expressed protein by routine procedures. In addition, whether the recombinant protein is expressed or not, which can be conveniently determined by fluorescence microscopic imaging with a standard fluorescence microscope. The strategy described in this study greatly simplifies the monitoring of dynamic changes in target protein expression during fermentation.

## Materials and methods

### Bacterial strains and agents

The *E. coli* strains and plasmids used in this study are shown in Table 1. *E. coli* Top10 was used for all the cloning steps, while BL21(DE3)pLysS and Rosetta(DE3)pLysS were used for recombinant protein expression. Restriction enzymes, Pyrobest DNA polymerase, dNTP and gel extraction kits were obtained from TaKaRa (Dalian, China). Isopropyl-β-d-thiogalactopyranoside (IPTG), and antibiotics were purchased from Sangon Biotech (Shanghai, China). Tryptone and yeast extract were obtained from OXIOD (Basingstoke, UK). *E. coli* was cultured in Luria–Bertani (LB) broth (1% tryptone, 0.5% yeast extract and 1% NaCl) supplemented with antimicrobial agents, as necessary.

**Table 1:** Bacterial strains and plasmids used in this study

	Genotype/characteristics	Source
Strains		
Top10	F^−^ *mcr*A Δ(*mrr*-*hsd*RMS-*mcr*BC) φ80*lac*ZΔM15 Δ*lac*X74 *rec*A1 *ara*D139 Δ(*ara*-*leu*)7697 *gal*U *gal*K *rps*L (Str^R^) *end*A1 *nup*G	Invitrogen
BL21(DE3)pLysS	F^−^ *ompT hsdS*_*B*_(*r*_*B*_^−^ *m*_*B*_^−^) *gal dcm* (DE3) pLysS (Cm^R)^	Novagen
Rosetta(DE3)pLysS	F^−^ *ompT hsdS*_*B*_(*r*_*B*_^−^ *m*_*B*_^−^) *gal dcm lacY1* (DE3) pLysSRARE (Cm^R^)	Novagen
Plasmids		
pET-21a	Amp^r^ T7 promoter lac operator	Novagen
pUCm-T	TA cloning	Sangon
pT-RFP	pUCm-T carrying *mcherry*	This study
pET-RFP	pET-21a expressing mCherry	This study
pO-RFP	pET-21a carrying *mcherry* in ORF2	This study
pOI-RFP	pO-RFP with one rare codon in ORF1	This study
pOII-RFP	pO-RFP with two consecutive rare codons in ORF1	This study
pOIII-RFP	pO-RFP with three consecutive rare codons in ORF1	This study
pT-GFP	pUCm-T carrying *egfp*	This study
pGFP-RFP	pO-RFP carrying *egfp* in ORF1	This study
pGFPI-RFP	pGFP-RFP with one rare codon preceding *egfp*	This study
pGFPII-RFP	pGFP-RFP with two consecutive rare codons preceding *egfp*	This study

### Construction of recombinant plasmids

DNA encoding red fluorescent protein mCherry (GenBank Accession No. MH070102) was optimized based on the *E. coli* codon bias and synthesized by Sangon Biotech (Shanghai, China). The synthesized fragment (708 bp) was cloned into pUCm-T to generate pT-RFP. All primers used in this study are shown in Table 2. The gene encoding mCherry was amplified by PCR from pT-RFP using the 1mF and 1mR primers, digested with *Nde*I and *Bam*HI, and then ligated into similarly digested pET-21a to yield pET-RFP, which produced recombinant mCherry under the control of T7 lac promoter. A 0.72 kb DNA fragment containing *mcherry* was amplified from pT-RFP using the 2mF and 2mR primers, then digested and ligated into the *Hin*dIII-*Xho*I sites of pET-21a, forming pO-RFP. DNA fragments (about 0.75 kb) were amplified from pO-RFP using the 2m1F/2m2F/2m3F and 2mR primers, then digested and inserted in pO-RFP via *Eco*RI and *Xho*I restriction sites, generating pOI-RFP/pOII-RFP/pOIII-RFP.

**Table 2:** Primers used in this study

Primer	Sequence 5′–3′	Restriction site
1mF	GGATCTGCATATGGTCTCTAAAGGCGAGGAAG	*Nde*I
1mR	CGGGATCCTTATTTGTACAGTTCGTCCATG	*Bam*HI
2mF	CCCAAGCTTAAGAAGGAGATATACATATG GTCTCTAAAGGCG	*Hin*dIII
2mR	CCGCTCGAGTTATTTGTACAGTTCGTCCATG	*Xho*I
2m1F	GGAATTC**CTA**GAGCTCCGTCGACAAG	*Eco*RI
2m2F	GGAATTC**CTAATA**GAGCTCCGTCGACAAG	*Eco*RI
2m3F	GGAATTC**CTAATAAGG**GAGCTCCGTCGACAAG	*Eco*RI
2eF	CGGGATCCATGGTAAGCAAGGGTGAG	*Bam*HI
2eR	CCCAAGCTTCTTTGTACAGTTCATCCATG	*Hin*dIII
2e1F	CGGGATCCATG**CTA**GTAAGCAAGGGTGAG	*Bam*HI
2e2F	CGGGATCCATG**CTAATA**GTAAGCAAGGGTGAG	*Bam*HI

Rare codons are represented in bold, and restriction enzyme sites are indicated with underline

The gene of eGFP (GenBank Accession No. MH070103) was synthesized according to the codon usage bias of *E. coli*, and subcloned into pUCm-T to generate pT-GFP. A 0.73 kb DNA fragment containing *egfp* was amplified from pT-GFP using the 2eF and 2eR primers, then digested and ligated into the *Bam*HI–*Hin*dIII sites of pO-RFP, forming pGFP-RFP. DNA fragments (about 0.73 kb) were amplified from pGFP-RFP using the 2e1F/2e2F and 2eR primers, then digested and inserted in pGFP-RFP via *Bam*HI and *Hin*dIII restriction sites, generating pGFPI-RFP/pGFPII-RFP. All recombinant plasmids were sequenced (Sangon Biotech, China) to confirm accordance with the design.

### Protein expression

*Escherichia coli* expression host BL21(DE3)pLysS or Rosetta(DE3)pLysS was transformed with recombinant vectors using a CaCl_2_-mediated transformation method (Hui et al. 2018a). The transformed *E. coli* cells were spread on LB agar plates containing 50 μg/mL ampicillin and 34 μg/mL chloramphenicol, and then cultured overnight at 37 °C.

A single colony was used to inoculate 3 mL of LB medium in a 15 mL conical tube supplemented with the above antibiotics, and cultured overnight in a 37 °C shaking incubator at 200 rpm. For optimal protein expression experiment, the pre-cultured recombinant *E. coli* as described above was diluted to an OD_600_ of 0.01 in 100 mL fresh LB broth containing antibiotics in 250 mL triangular flasks. The culture was grown at 37 °C for 3 h with rotation at 220 rpm, and the bacteria reached log phase with an optical density at 600 nm of 0.4–0.5. The cultures were then induced for 6 h with 0.5 mM IPTG and incubated at 37 °C with shaking at 220 rpm. The 5 mL cultures were sampled at regular intervals after induction, and OD_600_ was measured by using an iMark microplate reader (Bio-Rad, USA).

### Fluorescence quantitative analysis

The fluorescence intensity of eGFP or mCherry produced in *E. coli* was measured with Lumina fluorescence spectrometer (Thermo, USA). A 3-mL aliquot of *E. coli* culture or diluent was added to 1-cm low fluorescence background quartz cuvette. To test the fluorescence intensity of recombinant proteins, the excitation wavelength was set at 488 nm and the intensity of emitted fluorescence of eGFP at 507 nm (Tsien 1998) was recorded. Subsequently, the excitation wavelength was set at 587 nm and the intensity of emitted fluorescence of mCherry at 610 nm (Shaner et al. 2004) was recorded at the same time. The fluorescence intensity was normalized by dividing the fluorescence intensity at the emission wavelength of 507 nm (eGFP) or 610 nm (mCherry) by the OD_600_ value of the same sample.

### Fluorescence microscopy

An aliquot of 50 μL of *E. coli* culture was directly mounted onto a glass slide, and air-dried. *E. coli* cells were visualized using a Nikon Eclipse Ni fluorescence microscope coupled to a Nikon DS-Ri2 digital camera (Tokyo, Japan). A FITC filter (excitation at 475–490 nm and emission at 505–535 nm) was used to detect eGFP fluorescence, and a TRITC filter (excitation at 545–565 nm and emission at 580–620 nm) was used to detect mCherry fluorescence in *E. coli* cells. Representative images were captured using NIS Elements software (Nikon, Japan).

## Results

### Construction of a series of dicistronic vectors

**Fig. 1:**
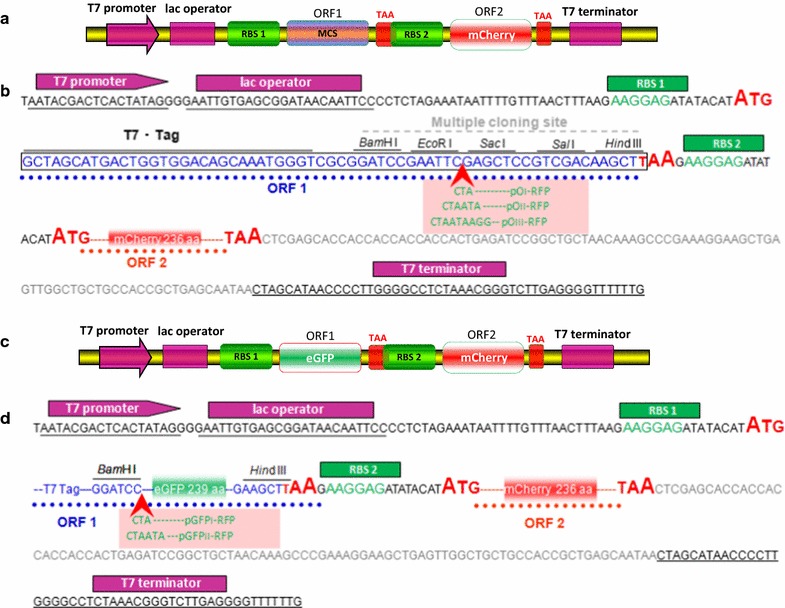
Experimental design and DNA sequence of the dicistronic constructs used in this study. **a** Schematic diagram of the dicistronic expression vector pO-RFP. RBS, ribosome-binding site; MCS, multiple cloning sites; ORF, open reading frame. **b** pO-RFP cloning/expression region. One/two/three consecutive rare codons were introduced into ORF1 of pO-RFP after *Eco*RI site, generating pOI-RFP/pOII-RFP/pOIII-RFP vector. **c** Schematic diagram of the dicistronic expression vector pGFP-RFP. The DNA fragment containing the eGFP coding sequence was directly ligated in pO-RFP previously digested with *Bam*HI and *Hin*dIII. **d** pGFP-RFP cloning/expression region. One/two consecutive rare codons were introduced into ORF1 of pGFP-RFP after *Bam*HI site, generating pGFPI-RFP/pGFPII-RFP vector

The strategy used in this study to construct the dicistronic vectors is shown in Fig. 1. The DNA fragment containing extra strong RBS, ATG initiation codon and the mCherry coding sequence was directly ligated in pET-21a previously digested with *Hin*dIII and *Xho*I, forming a dicistronic construct pO-RFP, which produced mCherry in the second ORF (ORF2). One/two/three consecutive rare codons were introduced following *Eco*RI site in the first ORF (ORF1) based on the PCR method, then generating pOI-RFP/pOII-RFP/pOIII-RFP, respectively.

The DNA fragment containing the eGFP coding sequence was directly inserted into ORF1 of pO-RFP to yield a dicistronic construct pGFP-RFP, which produced the target protein eGFP in ORF1 and the reporter protein mCherry in ORF2. One/two consecutive rare codons were placed upstream of *egfp* based on the PCR method, then generating pGFPI-RFP/pGFPII-RFP, respectively.

### mCherry expression in ORF2 was tuned by rare codons in ORF1

The log phase cultures of BL21(DE3)pLysS harboring pET-RFP, pO-RFP, pOI-RFP, POII-RFP, and pOIII-RFP were induced for 6 h with 0.5 mM IPTG at 37 °C, and there was no significant difference in the slope of the growth curve in these five recombinant *E. coli*. The OD_600_ of the cultures increased continuously since initiation of induction, and nearly reached a stable state after 4 h (data not shown).

**Fig. 2:**
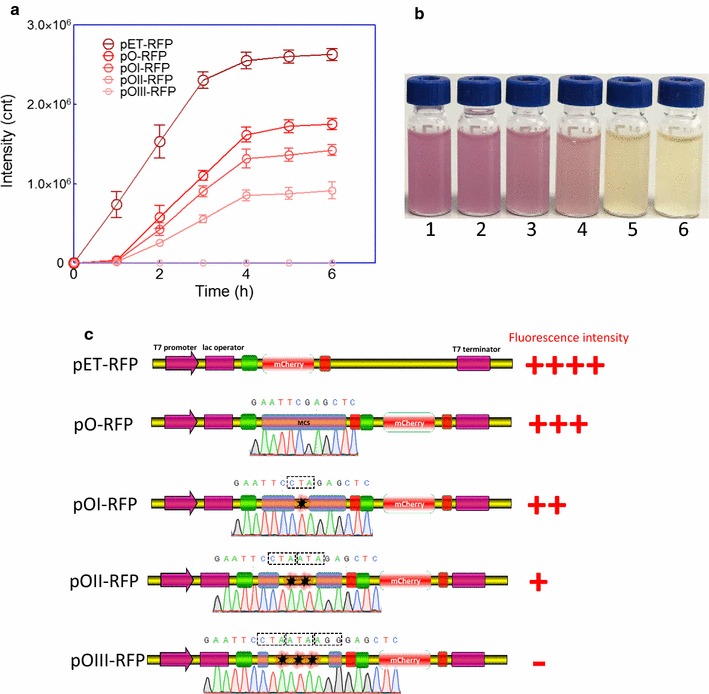
Effect of rare codons in ORF1 on mCherry expression in ORF2 in *E. coli* strain BL21(DE3)pLysS. **a** BL21(DE3)pLysS harboring various plasmids were grown for 6 h in LB media after 0.5 mM IPTG induction. mCherry fluorescence of bacterial cells suspension was measured every hour using a Luminar fluorescence spectrometer. The fluorescence intensity is indicated as a fluorescence count value (unit = cnt) and normalized by OD_600_. Data are representative of three independent experiments and values are expressed as mean ± SEM. **b** The recombinant *E. coli* suspension after 0.5 mM IPTG induction for 6 h. BL21(DE3)pLysS harboring different plasmids, 1. pET-RFP; 2. pO-RFP; 3. pOI-RFP; 4. pOII-RFP; 5. pOIII-RFP; 6. Control, BL21(DE3)pLysS harboring pET-21a with 0.5 mM IPTG induction. **c** Relationship of the genetic organization of recombinant plasmids and the expression level of the reporter gene *mcherry*

In the dicistronic vector pO-RFP, a short ORF1 composed of T7 Tag coding sequence and MCS, encoding a 22-amino acid peptide (the amino acid sequence of encoding polypeptide is MASMTGGQQMGRGSEFELRRQA), was placed upstream of ORF2 encoding mCherry. Compared with BL21(DE3)pLysS/pET-RFP, insertion of upstream short ORF1, resulted in significant decrease of mCherry expression (Fig. 2a). Although about 1 h mCherry expression lag resulted from the inserted short ORF1 existed, no significant improvement in mCherry expression was observed in all recombinant *E. coli* after 4 h induction (Fig. 2a). By changing the amount of rare codons inserted into ORF1, we sought to change the translation level of ORF1 and subsequently affect mCherry expression in ORF2. As expected, the introduction of rare codons in ORF1 lead to suppression of mCherry expression in ORF2, which was reflected by decreased fluorescence intensity throughout the induction time course (Fig. 2a). mCherry overexpressed in *E. coli* made the cultures light up in red, and color difference at 6 h terminal point was significantly obvious among these groups (Fig. 2b). The number of rare codons in ORF1 was negatively correlated with the expression of mCherry in ORF2 (Fig. 2c). Interestingly, three consecutive rare codons placed in ORF1 effectively abolished the expression of mCherry.

### Tuning mCherry expression in ORF2 with rare codons in ORF1 was partially recovered by Rosetta(DE3)pLysS host

The expression of gene containing rare codons can be dramatically improved when the cognate tRNAs are increased within the host (Mahalik et al. 2014). The Rosetta strains contain vector pRARE, which encodes the rare tRNA genes (Madje et al. 2012; Nguyen et al. 2011). Thus, the rare codons CUA, AUA, and AGG involved in pOI-RFP/pOII-RFP/pOIII-RFP should all be supplemented. When the host was transferred from BL21(DE3)pLysS to Rosetta(DE3)pLysS, the expression of ORF1 containing rare codons was expected to be recovered due to the supply of rare tRNAs.

**Fig. 3:**
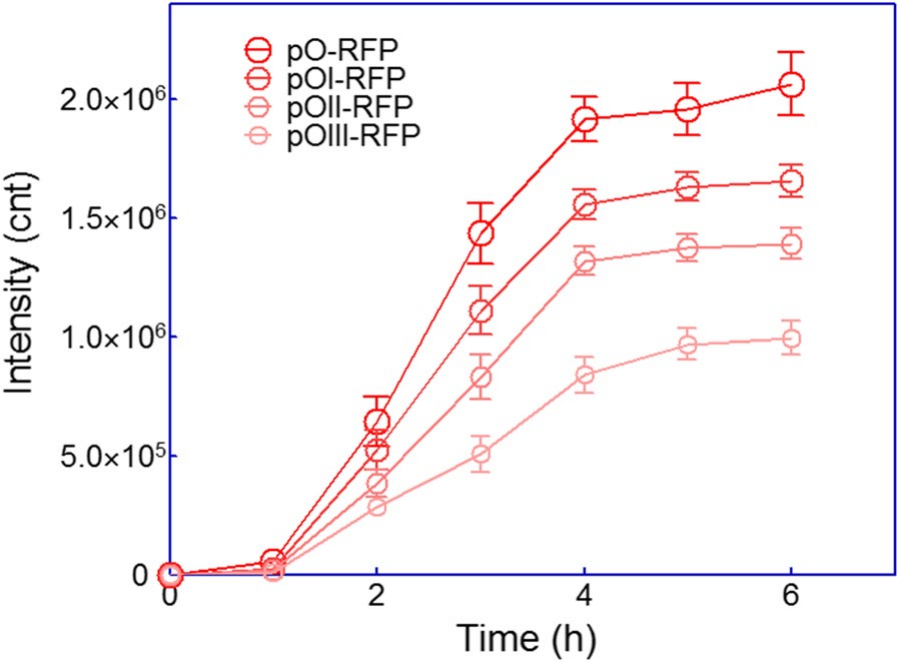
Effect of rare codons in ORF1 on mCherry expression in ORF2 in *E. coli* strain Rosetta(DE3)pLysS. Rosetta(DE3)pLysS harboring various plasmids were grown for 6 h in LB media after 0.5 mM IPTG induction. mCherry fluorescence of bacterial cells suspension was measured every hour using a Luminar fluorescence spectrometer. Fluorescence intensity values were normalized using the absorbance at 600 nm. Data are representative of three independent experiments and values are expressed as mean ± SEM

The log phase cultures of Rosetta(DE3)pLysS harboring four dicistronic plasmids, including pO-RFP, pOI-RFP, pOII-RFP, and pOIII-RFP, were induced for 6 h with 0.5 mM IPTG at 37 °C. The growth curve of recombinant Rosetta(DE3)pLysS was similar with that of recombinant BL21(DE3)pLysS, and there was no significant difference among four recombinant bacteria strains (data not shown). Although there were still significant differences in the red fluorescence intensity among four recombinant Rosetta(DE3)pLysS strains, obvious restored expression of mCherry in ORF2 was observed especially in Rosetta(DE3)pLysS/pOIII-RFP. The results are shown in Fig. 3.

### eGFP expression in ORF1 was positively correlated with mCherry expression in ORF2

The dicistronic vector pO-RFP can be assembled by cloning target gene into the MCS located in ORF1. In order to test whether the expression of target gene in ORF1 can be indicated by the fluorescence reporter mCherry, the model protein eGFP coding sequence as the target gene was subsequently inserted into the MCS, substituting for the original short ORF1. Because there is no overlap of the excitation and emission spectra of mCherry and eGFP, it is convenient to detect the expression of mCherrry and eGFP simultaneously by a fluorescence method (Shaner et al. 2004; Tsien 1998).

**Fig. 4:**
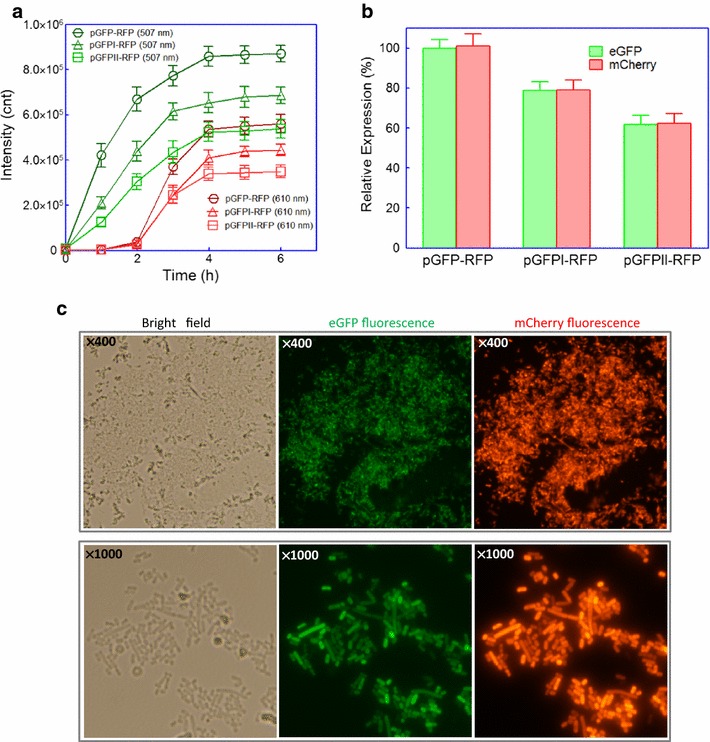
Relationship of the expression level of the target gene *egfp* in ORF1 and the reporter gene *mcherry* in ORF2. **a** Time course of the fluorescence intensity of eGFP and mCherry. BL21(DE3)pLysS harboring pGFP-RFP, pGFPI-RFP, and pGFPII-RFP were grown for 6 h in LB media after 0.5 mM IPTG induction. **b** Comparison of the co-expression levels of eGFP and mCherry in three recombinant *E. coli* at 6 h after IPTG induction. The fluorescence values of eGFP and mCherry in pGFP-RFP were defined as 100%, and the fluorescence values in pGFPI-RFP and pGFPII-RFP were normalized by dividing the actual fluorescence values by the fluorescence values of eGFP and mCherry in pGFP-RFP. Data are representative of three independent experiments and values are expressed as mean ± SEM. **c** Fluorescence microscopic analysis of *E. coli* BL21(DE3)pLysS/pGFP-RFP after 4 h induction with 0.5 mM IPTG. *E. coli* cells were visualized using a fluorescence microscope (×400 or ×1000 magnification). The fluorescence imaging of eGFP upon excitation at 475–490 nm and emission detection between 505 and 535 nm, and fluorescence imaging of mCherry upon excitation at 545–565 nm and emission detection between 580 and 620 nm. The same setting was applied for imaging of both eGFP and mCherry. At least three independent preparations were analyzed and representative images are shown here

The log phase cultures (OD_600_ = 0.4–0.5) of BL21(DE3)pLysS harboring pGFP-RFP, pGFPI-RFP, and pGFPII-RFP were induced with 0.5 mM IPTG at 37 °C, and the different kinds of dicistronic plasmids exerted no obvious effects on the growth of recombinant *E. coli* after induction (data not shown). Green fluorescence in all three recombinant bacterial cultures could be detected immediately at the early stage of induction, but there was about 1 h lag of mCherry expression after IPTG induction. No significant increase in both eGFP and mCherry expression was observed in all recombinant *E. coli* after 4 h induction (Fig. 4a). Rare codons placed near the N-terminus of the well-expressed protein eGFP were demonstrated to downregulate eGFP expression effectively, which is reflected in the eGFP fluorescence intensity (Fig. 4a). Interestingly, followed by downregulation of eGFP expression in ORF1, the decrease of mCherry expression in ORF2 was also observed. Expression level of the target protein eGFP could be reflected by red fluorescence intensity directly during induction (Fig. 4a) or at the end of induction (Fig. 4b). In addition, bacterial whole cell fluorescence imaging could be done based on both the target protein eGFP and the reporter protein mCherry. *E. coli* cells were conveniently visualized using a fluorescence microscope, and the fluorescence intensity in a single bacterial cell factory could be distinguished under 1000 times magnification (Fig. 4c).

## Discussion

In *E. coli*, RBS and other regulatory RNA sequences are essential control elements for translation initiation (Carrier and Keasling 1999; Isaacs et al. 2004). Translation initiation, the rate-limiting step of protein expression, is determined by several molecular interactions, such as the complementarity of the RBS sequence to 16S rRNA, the spacing between RBS and the start codon, and the presence of mRNA secondary structures (Salis et al. 2009). In some cases, the distance between the promoter and structural genes in natural operons is 60 bases above. The necessary elements for translation initiation including: the standby site, 16S rRNA binding site, spacing and the start codon are all included in the intergenic regions. So each gene in the polycistronic mRNA strand can be translated independently and tunably (de Smit and van Duin 2003; Jacob et al. 2005; Kudla et al. 2009).

The underlying goal of synthetic biology is to develop concise and standardized biological parts, so no redundant sequences are retained (Shetty et al. 2008). How do we ensure that the downstream genes in the artificial operon can be translated successfully? The Shine–Dalgarno (SD) sequences are obviously necessary but not sufficient. Several effective strategies were developed to address this issue, including: a large fragment of optimal or natural DNA sequence containing the elements for the translation initiation was placed in the intergenic regions (Wang et al. 2015; Yadav et al. 2014); the coding sequence or the stop codon of the upstream gene was overlapped with the SD sequence of the downstream gene (Ito and Kurosawa 1992; Palleja et al. 2009); and the SD sequence of the downstream gene was placed immediately after the termination codon of the upstream gene (Esipov et al. 2016; Gaymard et al. 2000).

**Fig. 5:**
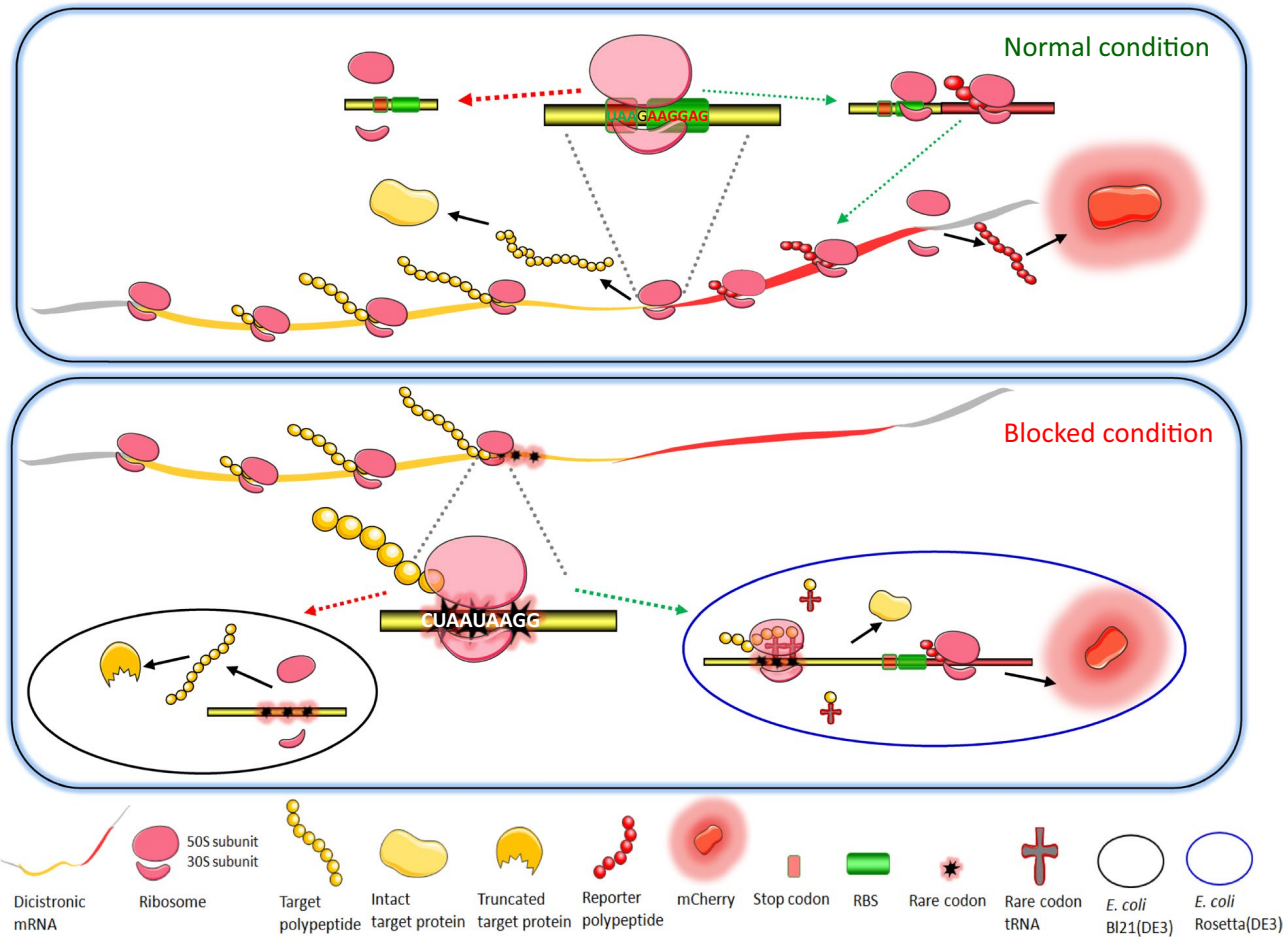
A dicistronic construction-based fluorescence indication of the target gene expression

In order to achieve the co-regulation of structural genes, functionally related genes that are common in both prokaryotic and eukaryotic genome are often found in close proximity (Gasch and Eisen 2002; Palleja et al. 2009). Inspired by this phenomenon, previous studies have demonstrated that the expression of structural genes could be co-regulated in an artificial operon by assembling them in tandem in close proximity, and high expression was always achieved in the upstream gene (Cheng et al. 2016; Esipov et al. 2016; Wang et al. 2015). The distance between the stop codon of the upstream gene and the start codon of the downstream gene was demonstrated to be of key importance in translational coupling (Levin-Karp et al. 2013). These findings suggest that translation of co-regulated structural genes was initiated not only in order but also coupled together. To develop a simple approach for indicating the target protein expression of *E. coli* in real-time, we engineered a short ORF containing MCS for inserting the target gene, which was placed upstream of another ORF encoding the reporter fluorescence protein. To achieve a high-level co-expression of reporter mCherry with the target gene, an optimized strong RBS and spacing between SD sequence and ATG initiator codon (Pfleger et al. 2006) were placed immediately after the termination codon of the first ORF. Molecular mechanism of the hypothesis involved in this study is shown in Fig. 5. It allows the two genes to be co-transcribed into one dicistronic mRNA strand under the control of T7 lac promoter, and not only independent but also coupling production of target protein and reporter. No extra or enough ribosomes might bind RBS2 directly, because not enough characteristic DNA sequences related to translation initiation were included in the short length between the target gene and the report gene. During normal conditions when the ribosome moves to the first terminal codon, nascent target polypeptide will be released. However, the ribosome tends to dissociate. Due to the next RBS immediately following the terminal codon, a few residual ribosomes that have not yet dissociated will continue to initiate the expression of the reporter. During the blocked condition, a variety of possible factors, such as consecutive rare codons in the first ORF, may lead to failure in target gene expression. Most stalled ribosome will then be subsequently released due to tRNA non-availability, and the mCherry reporter expression will be closed. Another important mechanism involved in the coupled translation of tandem cistrons might be that highly efficient polycistronic mRNA degradation is always triggered by substrate starvation, depletion of nutritional elements, etc. (Mahalik et al. 2014). The dicistronic mRNA degradation could be conveniently monitored using real time PCR during blocked conditions.

Rare codons generally influence the rate and efficiency of translation, due to rarity of their cognate tRNAs (Kane 1995; Parmley and Huynen 2009; Rosenberg et al. 1993). Protein expression could be regulated precisely by changing the combination of genetic codes in both mammalian cell and engineered bacterial cells (Brule and Grayhack 2017; Wang et al. 2016). The rare codons involved in this study (CTA, ATA and AGG) were used at a frequency of < 0.5% in *E. coli*, and previous studies have demonstrated that the above rare codon alone could cause translational problems in *E. coli* (Chumpolkulwong et al. 2006). The translation efficiency of short ORF1 in pO-RFP was adjusted successfully using the inserted rare codons, and it was well reflected indirectly from the fluorescent signal derived from the downstream ORF2. Interestingly, due to the introduction of three consecutive rare codons into the upstream short ORF, the translation of mCherry in the downstream ORF was shut down in BL21(DE3)pLysS, but was partially recovered in Rosetta(DE3)pLysS (rare tRNAs were supplemented). Furthermore, the model protein eGFP was inserted into the upstream ORF1, and various expression levels of eGFP were obtained by varying rare codons preceding *egfp*. Importantly, a positive correlation was found between the expression levels of eGFP in ORF1 and mCherry in ORF2.

Fluorescence proteins have been successfully overexpressed in a wide range of organisms without obvious cytotoxic effects, and they have great potential as reporters for monitoring gene expression (Cubitt et al. 1995). In this study, no inclusion bodies were formed during over expression of mCherry in BL21(DE3)pLysS/pET-RFP (data not shown). Growth retardation and bacterial cell disruption were sometimes observed when recombinant proteins were expressed at high levels (Mahalik et al. 2014). Interestingly, red fluorescent signal was also detected in culture supernatant of BL21(DE3)pLysS/pET-RFP at the late phase of IPTG induction (data not shown). This suggests that the integrity of microbial cell factories could be checked during fermentation by determination of the fluorescence in culture supernatant.

Based on the strategy mentioned in this study, the fluorescent signal can be noninvasively detected in living bacterial cells during fermentation in real time, and it eliminates the analysis of exogenously expressed protein by routine procedures. There are still some shortcomings in the dicistronic system in this study. First of all, although no mCherry expression lag was observed in BL21(DE3)pLysS/pET-RFP, slight mCherry expression lag (about 1 h) still existed in the dicistronic vectors in this study. Compared to an enhanced GFP variant (eGFP), mCherry matures quickly at 37 °C with maturation half time of 15 min (Shaner et al. 2004). Previous studies showed both mCherry and eGFP could rapidly fold and mature even when fused to poorly folding polypeptides (Aper et al. 2014; Tsien 1998). There was not enough evidence showing that 1 h lag of fluorescent signal resulted from incomplete maturation of mCherry. The expression of two ORFs involved in the dicistronic vectors in the study might be initiated in order, so there was a time lag between translation initiation of ORF1 and ORF2. In addition, most ribosomes dissociated at the stop codon of ORF1, followed by a great decrease in the expression of mCherry in ORF2. Due to the reasons above, the accumulation of matured mCherry might be below the detection limit at the early stage of induction. However, it hardly exerted any influence on the result after 2 h induction. More importantly, a 4–6 h induction was generally adopted in most protein expression studies. Second of all, it is inevitable that the synthesis of reporter mCherry in downstream ORF2 may lead to an unnecessary metabolic burden on the cell. Extra substrate and energy consumption might exert a negative influence on the yield of highly expressed target protein in ORF1, but the influence could also become insignificant when a poorly expressed target gene is inserted in ORF1. To decrease the uncertainties of the influence on the production of the target protein, a low molecular weight and easy detectable reporter might be a good solution.

## Abbreviations

*E. coli*: *Escherichia coli*
LB: Luria–Bertani
RFP: red fluorescent protein
GFP: green fluorescent protein
IPTG: isopropyl-β-d-thiogalactopyranoside
RBS: ribosome-binding site
MCS: multiple cloning sites
ORF: open reading frame
SDS-PAPE: sodium dodecyl sulfate polyacrylamide gel electrophoresis
ELISA: enzyme linked immunosorbent assay
kDa: kilodalton
SD: Shine–Dalgarno
